# Developing psychological treatments for psychosis

**DOI:** 10.1192/bjp.2024.5

**Published:** 2024-05

**Authors:** Daniel Freeman

**Affiliations:** Department of Experimental Psychology, University of Oxford, UK; and Oxford Health NHS Foundation Trust, UK

**Keywords:** Psychosocial interventions, psychotic disorders/schizophrenia, clinical trials, community mental health teams, mental health services

## Abstract

Evidence shows that talking with patients about psychotic experiences can be beneficial. The key question is therefore: which psychological methods can help patients most? This editorial presents ten principles for the design and development of effective psychological treatments. These principles are perceptible characteristics of successful interventions.

People with psychosis often experience multiple and severe difficulties. Their psychological well-being is frequently in the lowest percentiles of the general population. Their physical health tends to be poorer too. It is therefore vitally important that treatments can deliver large clinical benefits. The first wave of cognitive–behavioural therapy for psychosis has shown that talking about psychotic experiences with patients is safe, popular and leads to modest clinical benefits.^1^ It points the way towards more powerful psychological approaches. Much improved outcomes for one psychotic symptom, persecutory delusions, have been delivered by a new theory-driven psychological treatment (the Feeling Safe programme) developed over a decade of research.^2^ Feeling Safe was shown to bring benefits beyond those that come with a positive therapeutic relationship. In this editorial I set out the design principles underpinning this successful intervention, which become attributes of the treatment manifest to patients and therapists alike. These principles may be of value in the development of future new treatments.

## Be respectful

Showing respect is the *sine qua non* of successful psychological therapies. In face-to-face treatment respect is the foundation of the therapeutic alliance that is so important for successful outcomes.^3^ The quality of respect – and warmth too^4^ – should be an essential aspect of digital treatments also. Respect in treatment delivery should be obvious to patients, who so often experience the very opposite in their lives. But respect is not simply a matter of delivery. It must be built into the treatment itself. For example, development must start from an understanding of patients’ likely difficulties, routes to change and perception of the intervention. The treatment must be sufficiently flexible to accommodate inevitable differences in patients’ readiness, ways of learning and preferences. Facilitation of patient choice, and a thoughtfulness and transparency in the presentation of information, are also important. The result should be a treatment that is empathic, focused on patient need and thus engaging to patients.

## Be precise

The diagnoses of psychosis arguably encompass the most varied and distinct symptoms of any mental health condition. Factor analytic studies suggest at least half a dozen distinct experiences within the diagnosis of schizophrenia alone, including paranoia, grandiosity, hallucinations, conceptual disorganisation, anhedonia and emotional expression difficulties. Common too are multiple co-occurring problems such as anxiety, depression, cognitive impairment and social functioning difficulties. The development of a new treatment must therefore proceed from a precise articulation of the key outcome target, mechanistic route to change and main therapeutic techniques. Such a highly specified approach can lead to a therapeutic focus and rigour that maximise the likelihood of positive change.

## Target key mechanisms

A successful treatment must target the key mechanisms underlying a difficulty. These are the main drivers of the difficulty; the mechanisms that, when altered, are likely to deliver most change. Such targeting requires a plausible, logical explanation of how the mechanism leads to the difficulty. Moreover, the mechanism must be tractable to psychological approaches. A focus on a key mechanism could of course bring about change in a number of different outcomes. Experimental work, often with people with non-clinical instances of the difficulty, can provide important support for the mechanistic focus.^5^ Desirable too are the inclusion of mediation tests in clinical trials to show that the treatment is acting as designed^6^ and examination of outcome trajectories and their predictors.

## Frame positive counterweights

The use of positive counterweights can be a highly effective means of generating therapeutic change. For example, because individuals with persecutory delusions typically feel unsafe a helpful framing of the treatment target is to feel safe. Similarly, because negative voices often provide uncontested warnings of danger the counterweight objective is for the person themselves to decide what is happening. Such framing does not directly challenge a patient's current viewpoint, which can have the unintended consequence of strengthening that position. Instead, it provides a pathway to positive change that can coexist with the current position until the new perspective becomes dominant.

## Allow for complexity

A danger of the mechanistic approach is that it can be overly simplistic. In truth, causation in psychosis is typically a complex, multifactorial matter. Each factor can be considered as an ‘inus condition’:^7^ that is, ‘an insufficient but non-redundant part of an unnecessary but sufficient condition’. A single factor only increases the probability of a difficulty occurring. It should be noted too that causation can vary in individual instances, with differences in both the combination of factors and each factor's influence. Moreover, patients may differ in their preferences regarding routes to change. All this means that treatments are more likely to be able to address individual clinical need if personalisation is included in the design.

## Build in measurement

Successful treatment relies on comprehensible, meaningful and accurate measurement of the key outcomes and mechanistic targets of treatment. Assessments, which can be shared with patients, are an essential resource for monitoring and directing treatment. Each clinical session should include a brief outcome assessment. If treatment is not going well, such measurement can inform a modified approach. The potential for negative effects of therapy also requires attention. In addition to the measurement of treatment outcomes, treatment delivery must be assessed for adherence and competency. The reality is that measurement of problems in psychosis is often outdated. Developing new interventions, therefore, typically means also devising new assessments.

## Involve those with lived experience

Consultation with people with lived experience must be an integral part of treatment design. The form of that consultation is likely to vary depending on the stage of the design process but may include surveys to assess need and preferences, qualitative studies to advance understanding of the difficulty, and early consideration of issues of inclusion. A patient advisory group is best formed as early as possible. It can then give guidance throughout the design and testing process. It can also be valuable to have a wider network of patients to consult. Patients often find it helpful to hear the stories of people who have undergone similar experiences. It is desirable to collect these accounts early in the design process and embed them in the intervention. The patient advisory group can play a key role in facilitating this collection of personal stories.

## Build credibility

Patients’ perception of treatment credibility is associated with better outcomes. It is therefore desirable to give patients an accurate picture of the evidence base for an intervention. Naturally, successful treatments require rigour at each stage of development. This includes both setting out the theory underpinning the intervention and clinical testing. Currently, proof-of-concept testing to show that an intervention is likely to have a large clinical effect is often relatively neglected in the race to a randomised controlled trial. Dose-finding studies are also rare in psychological therapy research. The foundations of the theory, and the results of early-stage testing, should be as robust as possible before moving to a lengthy efficacy trial.

## Develop optimism

Also associated with better outcomes is patients’ perception of treatment efficacy: expectancy. Assuming that the early-stage clinical testing is suitably promising, we can embed this optimism in the design of the intervention. This can take the form, for example, of a clear, concise explanation of outcome evidence, ideally including proportions of people who tend to benefit or not, and of why the intervention may be helpful. Optimism is possible when a treatment is shown to work well. Such optimism (and transparency) can counter the disillusionment and feelings of hopelessness that many patients who have been in services for years can experience.

## Plan for implementation

Typically in treatment development the aim is to maximise the treatment outcome effect and then later consider adaptations for wider use. The simplest – although hardly effort-free – approach to those adaptations is detailed manualisation of the intervention, associated training requirements, and supervision and monitoring processes. Such a strategy has merit, but ideally future implementation should be considered at the outset. For an efficacious intervention to be used at scale, a diverse workforce must be able to deliver or support it without weakening the clinical effect. Identifying barriers and facilitators at the earliest stages allows implementation factors to be built into the treatment design. A keen eye on implementation can also serve as a helpful reminder of the need for clarity, concision and focus in the intervention.

## Conclusion

A treatment that is respectful to patients, that targets key and tractable causal factors and that includes responsiveness to regular measurement will have a chance of proving efficacious. The ten design principles outlined above are relevant to all stages of treatment development, from design brief through to clinical testing and to implementation in practice. Rather than merely implicit, they are principles that patients and practitioners should be able to notice as they use the intervention. I hope they may help provide a route to the highly effective therapies that are so urgently needed by patients with psychosis.

## Data Availability

Data availability is not applicable to this article as no new data were created or analysed in this study.
